# Dependence on mitochondrial respiration of malignant T cells reveals a new therapeutic target for angioimmunoblastic T-cell lymphoma

**DOI:** 10.1038/s41420-024-02061-9

**Published:** 2024-06-19

**Authors:** Adrien Krug, Rana Mhaidly, Marie Tosolini, Laura Mondragon, Gamze Tari, Adriana Martinez Turtos, Rachel Paul-Bellon, Vahid Asnafi, Sandrine Marchetti, Léa Di Mascio, Marion Travert, Frédéric Bost, Emmanuel Bachy, Rafael J. Argüello, Jean-Jacques Fournié, Philippe Gaulard, François Lemonnier, Jean-Ehrland Ricci, Els Verhoeyen

**Affiliations:** 1 Université Côte d’Azur, INSERM, C3M, 06204 Nice, France; 2 Equipe labellisée Ligue Contre le Cancer, 06204 Nice, France; 3 CRCT, Université de Toulouse, Inserm, CNRS, Université Toulouse III-Paul Sabatier, Centre de Recherches en Cancérologie de Toulouse, Toulouse, France; 4 T cell lymphoma group, Josep Carreras Leukaemia Research Institute (IJC), Josep Carreras Building, Ctra de Can Ruti, Camí de les Escoles, s/n, 08916 Badalona, Spain; 5 Université Paris-Est Créteil; Institut Mondor de Recherche Biomédicale, INSERMU955; Unité hémopathies lymphoïdes, Hôpitaux Universitaires Henri Mondor, Assistance publique des Hôpitaux de Paris, Créteil, France; 6 Laboratory of Onco-Hematology, Institut Necker Enfants-Malades, Université Paris-Cité and INSERM U1151, Paris, France; 7 Hospices Civils de Lyon and Claude Bernard Lyon 1 University, Lyon, France; 8 Aix Marseille Univ, CNRS, INSERM, CIML, Centre d’Immunologie de Marseille-Luminy, Marseille, France; 9 Labex TOUCAN, Toulouse, France; 10 AP-HP, Groupe hospitalo-universitaire Chenevier Mondor, département de pathologie, F-94010 Créteil, France; 11 AP-HP, Groupe hospitalo-universitaire Chenevier Mondor, Service Unité Hémopathies Lymphoides, F-94010 Créteil, France; 12 CIRI, Université de Lyon; INSERM U1111; ENS de Lyon; University Lyon1; CNRS, UMR5308, 69007 Lyon, France

**Keywords:** Lymphoma, Cancer models

## Abstract

Cancer metabolic reprogramming has been recognized as one of the cancer hallmarks that promote cell proliferation, survival, as well as therapeutic resistance. Up-to-date regulation of metabolism in T-cell lymphoma is poorly understood. In particular, for human angioimmunoblastic T-cell lymphoma (AITL) the metabolic profile is not known. Metabolic intervention could help identify new treatment options for this cancer with very poor outcomes and no effective medication. Transcriptomic analysis of AITL tumor cells, identified that these cells use preferentially mitochondrial metabolism. By using our preclinical AITL mouse model, mimicking closely human AITL features, we confirmed that T follicular helper (Tfh) tumor cells exhibit a strong enrichment of mitochondrial metabolic signatures. Consistent with these results, disruption of mitochondrial metabolism using metformin or a mitochondrial complex I inhibitor such as IACS improved the survival of AITL lymphoma-bearing mice. Additionally, we confirmed a selective elimination of the malignant human AITL T cells in patient biopsies upon mitochondrial respiration inhibition. Moreover, we confirmed that diabetic patients suffering from T-cell lymphoma, treated with metformin survived longer as compared to patients receiving alternative treatments. Taking together, our findings suggest that targeting the mitochondrial metabolic pathway could be a clinically efficient approach to inhibit aggressive cancers such as peripheral T-cell lymphoma.

## Introduction

Adenosine triphosphate (ATP) is the key energy molecule produced by glycolysis and oxidative phosphorylation (OXPHOS) to fulfill the bioenergetic needs of each cell. Glycolysis is the main anabolic reaction used by all cell types to generate energy for rapid growth and proliferation. Moreover, glycolysis allows the generation of metabolic intermediates, which can be used in other biosynthetic pathways necessary for cell growth. OXPHOS, or mitochondrial respiration is the energy power source of the cell because of its capacity to produce a more abundant amount of ATP as compared to glycolysis [1]. In normal conditions, healthy cells balance nutrient consumption and metabolism to successfully maintain functional integrity and the ability to divide [2, 3]. In contrast, malignant cells undergo metabolic reprogramming to meet their high energy requirements. Cancer cells preferentially utilize glucose via aerobic glycolysis, a process known as Warburg Effect [2]. However, our understanding of the complex metabolic reprogramming that cancer cells use to face stressful environmental conditions has advanced [1]. Recently, it has been shown that metabolic reprogramming toward OXPHOS frequently occurs in tumors [3, 4]. Importantly, many of these metabolic changes are controlled by co-inhibitory pathways, e.g., the axis of the PD-1/PD-L1 engaged by cancer cells in order to block immune cell functions [5]. Therefore, identifying and targeting the specific immune-metabolic modifiers might have significant clinical implications in cancers where co-inhibitory signals play a major role.

A rare peripheral T cells lymphoma (PTCL), called angioimmunoblastic T-cell lymphoma (AITL) is a devastating disease. AITL disease outcome is poor, with an overall 5-year survival rate of 30% upon chemotherapeutic treatment, and optimal management of the disease is yet to be defined. AITL is recognized as a CD4 T-cell disorder with a T follicular helper (Tfh) cell phenotype, associated with germinal center (GC) B cell dysregulation [6]. AITL Tfh cells show as their healthy Tfh counterparts high expression levels of the surface markers CXCR5, ICOS, and PD-1. They express the chemokine CXCL13, a B cell attractant, and Bcl-6, both important for Tfh differentiation [7]. Currently, the metabolic requirements of the Tfh AITL cells and the tumor microenvironment (TME) still need to be elucidated. The identification of metabolic pathways used by AITL cells and TME might allow us to propose new therapeutic strategies for these patients.

Previously, we established a new preclinical mouse model for AITL, by overexpressing the glycolytic enzyme GAPDH, exclusively in the T-cell lineage (plck-GAPDH mouse). By transcriptional profiling, genetic approaches, and immuno-phenotyping of the plck-GAPDH tumors, we demonstrated that this mouse model recapitulated multiple pathological and immune-phenotypic features of the human AITL disease [8, 9]. Interestingly, we confirmed the activation of the non-canonical NF-kB pathway in the malignant CD4+ T cells in this preclinical murine AITL model. Moreover, the induction of the NF-kB pathway was confirmed in hAITL neoplastic cells and blocking of the pathway increased mAITL mouse survival [8]. Recently, we demonstrated the dependance of malignant murine and human AITL T cells on choline lipid metabolism [10]. Through interference with the choline metabolic pathway, survival of the mAITL preclinical mice was increased. Both these studies underline that this is a valid preclinical model for the evaluation of new therapies.

Here, we analyzed the metabolic status of human AITL tumor cells and demonstrated their dependence on oxidative phosphorylation. PD-1^high^ malignant AITL cells in our murine AITL preclinical model also relied on oxidative phosphorylation (OXPHOS) for growth and survival. Furthermore, targeting mitochondrial respiration using inhibitors of complex I of the mitochondrial electron transport chain (ETC), resulted in significantly increased survival of mAITL lymphoma-engrafted mice. The complex I inhibiting effect was confirmed for hAITL biopsies, and in a retrospective study of diabetic patients suffering from PTCL, metformin treatment showed a marked benefit.

## Results

### Human AITL tumors rely on OXPHOS as the main energy source

**Fig. 1:**
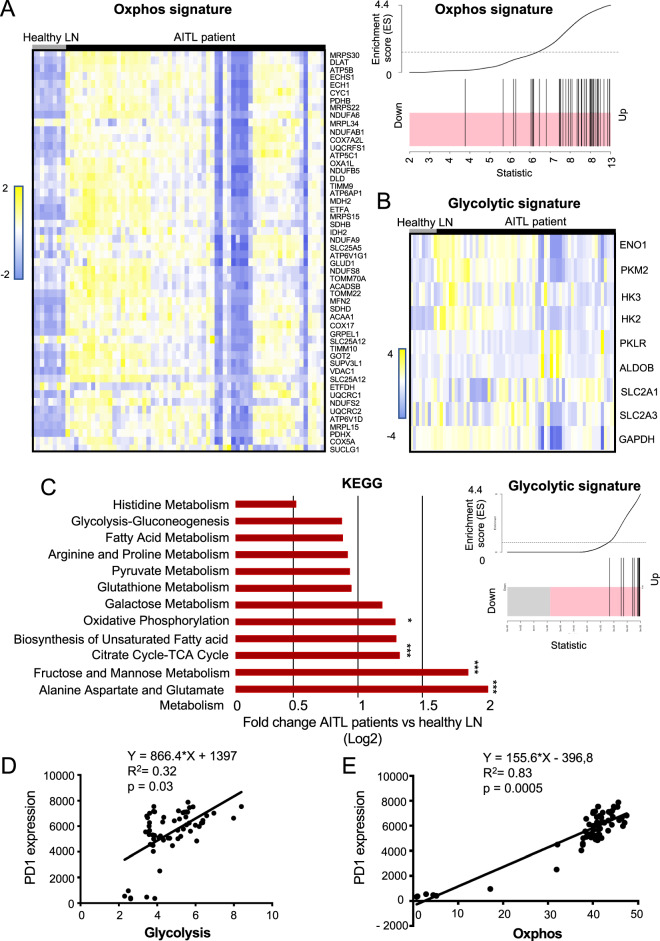
Human AITL tumors show a strong upregulation of oxidative phosphorylation signature genes (oxphos). **A** Heatmap for GSEA data of 52 genes (implicated in mitochondrial respiration (OXPHOS signature) for AITL patient (*n* = 60) and healthy LNs (*n* = 8). The corresponding GSEA for the OXPHOS signature genes indicated in **A** is shown at the right. For all genes with enrichment score >0 (black bars in the pink zone), expression is upregulated. Kolmogrov–Smimov (KS) test. **B** Heatmap for GSEA data for 9 genes implicated in glycolysis comparing AITL patient (*n* = 60) and healthy LNs (*n* = 8). The corresponding GSEA for the glycolytic signature genes indicated in **B** is shown under the heatmap. **C** Metabolic pathway analysis from RNAseq data from AITL patient lymphoma biopsies versus healthy lymph nodes was performed using the KEGG database. *p < 0.05; ***p < 0.001. **D** Correlation of PD-1 expression with a glycolytic gene expression signature or with an OXPHOS signature in hAITL tumors.

We have shown previously that the glycolytic function of GAPDH in the malignant T cells is not prominent in the end stage of AITL tumor development since inhibition of GAPDH enzymatic activity with its specific inhibitor, kongenic acid, did not prolong survival of our preclinical AITL mice [8]. Therefore, to decipher the metabolic pathways that mediate tumor cell survival in AITL, we compared gene expression data for lymph node (LN) biopsies from AITL patients [11, 12] to healthy LN. We performed gene set enrichment analysis (GSEA) for an OXPHOS signature [13] and observed a statistically significant enrichment in AITL samples for a set of 52 genes encoding for mitochondrial respiration enzymes (Fig. 1A) and ETC complexes (Fig. S1).

Using hallmark metabolic signatures, KEGG database analysis indicated that amino-acid metabolism and OXPHOS signatures were among the enriched pathways in hAITL tumors, while glycolysis did not show a marked upregulation (Fig. 1C). However, an upregulation was observed for some genes encoding for glycolytic enzymes and glucose transporters (Fig. 1B). It is known that Tfh PD-1^+^ cells are the drivers of AITL development and we showed that PD-1 mRNA expression presented a very weak correlation with a glycolytic gene signature, but a strong correlation with an OXPHOS gene signature (Fig. 1D, E), suggesting that PD-1 might act as a marker of OXPHOS in AITL tumor cells in agreement with Patoukis et al. [14].

### PD-1^high^ Tfh cells in the AITL mouse model rely on mitochondrial respiration

**Fig. 2:**
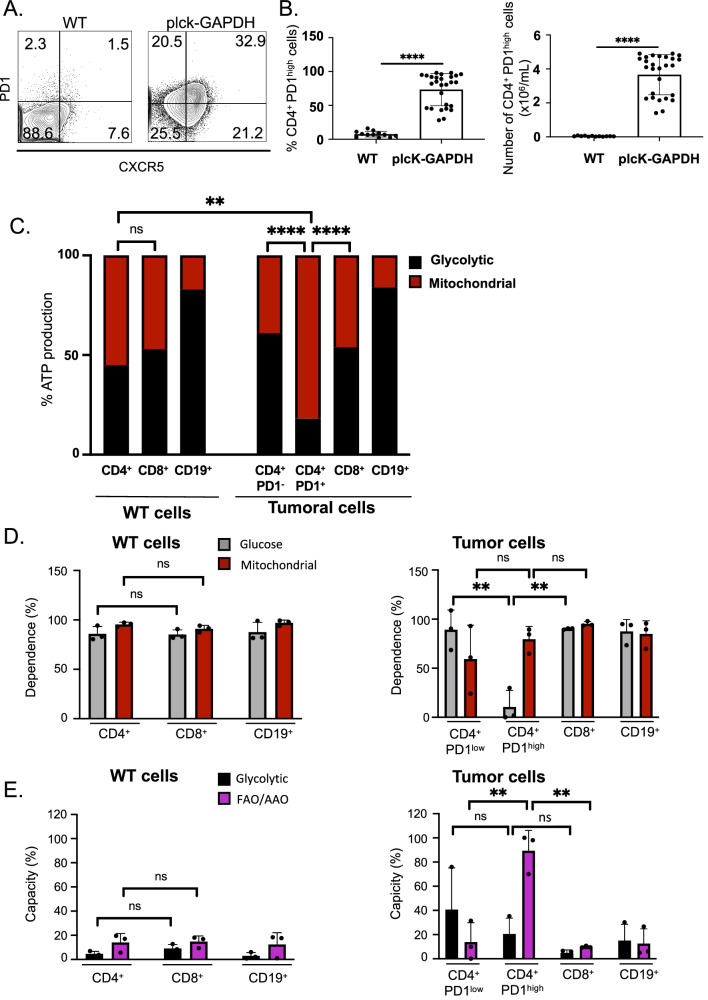
Plck-GAPDH malignant CD4+ PD-1^high^ cells are addicted to mitochondrial respiration. **A** FACS analysis of Tfh markers PD-1 and CXCR5 on CD4+ T cells in plck-GAPDH tumors compared to WT CD4+ splenocytes. **B** The percentage CD4+ PD-1^high^ cells in plck-GAPDH tumors compared to WT splenocytes per total CD4+ cells (mean ± SD, WT *n* = 12, plck-GAPDH, *n* = 27, ****p < 0.0001) and the number of CD4+ PD-1^high^ cells in plck-GAPDH lymphoma-bearing spleens and in WT spleens (mean ± SD, WT *n* = 12, plck-GAPDH, *n* = 27, ****p < 0.0001). **C** WT CD4+ splenocytes and plck-GAPDH tumor CD4+ and CD8+ T cells and CD19 + B cells were sorted by negative selection; the CD4+ tumor cells were further sorted for the PD-1^high^ fraction and PD-1 negative fraction). In each of these cell fractions we determined their mitochondrial and glycolytic metabolic ATP requirements using a method based on Cell Titer Glo Kit (mean is shown for *n* = 3; **p < 0.01, ****p < 0.0001). **D**, **E** PD-1, CD4, CD8, and CD19 surface antibody staining for WT splenocytes and plck-GAPDH tumors was followed by SCENITH metabolic FACS analysis to determine the dependance on glucose or mitochondria for the different cell subpopulations (**E**) and their glycolytic or fatty acid oxidation capacity (**E**) (mean ± SD, *n* = 3; **p < 0.01, ns not significant).

AITL is a very rare disease. Fortunately, we developed the preclinical murine AITL (mAITL) model, which mimics human AITL [8, 10]. Resembling human AITL disease, mAITL CD4^+^ tumor cells are indeed positive for Tfh markers (PD-1, CXCR5) (Fig. 2A, B).

Equivalent to human AITL LN biopsies, we observed in mAITL lymphomas, as compared to WT splenocytes, a significant upregulation for the expression of 52 genes implicated in mitochondrial respiration or an ECT gene signature (Fig. S2A, B). Analysis of genes implicated in the glycolytic pathway showed variable enrichment in mAITL tumors compared to WT (Fig. S2C).

The pool of CD4^+^ T cells in the mAITL tumors contains two subsets: the PD-1^−^ cells and the PD-1^high^ cells, which are considered the neoplastic cells of AITL malignancy. We therefore tested whether PD-1 expression was correlated with the metabolic alterations observed in mAITL tumors. We isolated both PD-1^−^ and PD-1^high^ cells from mAITL tumors. Since the malignant mAITL CD4^+^ PD-1^high^ T cells are accompanied by B cells and CD8 T cells, we also isolated these from plck-GAPDH tumors and WT splenocytes. As expected, malignant mAITL CD4^+^ PD-1^high^ cells produced significantly more mitochondrial ATP than WT CD4+ T cells and their CD4^+^ PD-1^−^ counterparts (Fig. 2C). Tumoral and WT CD8 cells used as well mitochondrial as glycolytic ATP as energy source, while B cells isolated from mAITL tumors or WT B cells mainly relied on glycolysis (Fig. 2C).

However, cell isolation before metabolic analysis might induce metabolic switches in the cells of interest. Therefore, the above data were additionally evaluated by a simple method for complex metabolic profiling called SCENITH [15], which does not require physical separation of the lymphocyte subsets before metabolic analysis. Flow cytometry gating on the malignant CD4+ PD-1^high^ T cells showed their low dependence on glucose, and they were significantly more dependent on OXPHOS as compared to the CD4+ PD-1^low^ cells in the AITL tumor, WT CD4+, CD8+, and CD19+ cells and tumor-infiltrating CD19+ and CD8+ cells (Fig. 2D). CD4+ PD-1^high^ cells showed significantly higher fatty acid oxidation capacity in contrast to CD4+ PD-1^low^ mAITL cells or WT CD4 splenocytes (Fig. 2E), which we recently confirmed in Krug et al. [10]. We concluded that CD4+ PD-1^high^ cells from mAITL tumors rely on OXPHOS metabolism for energy production.

### Human AITL malignant Tfh cells rely preferentially on mitochondrial respiration

**Fig. 3:**
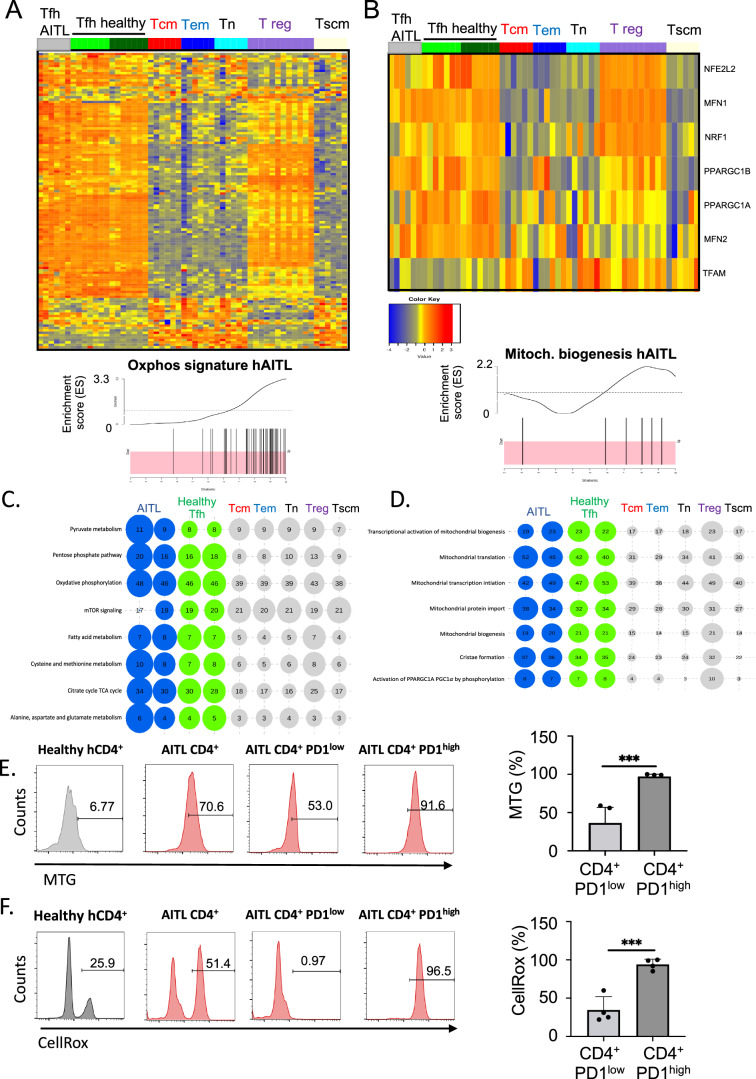
Human AITL malignant Tfh cells are addicted to mitochondrial respiration. **A** Heatmap for GSEA data for 52 genes implicated in mitochondrial respiration (OXPHOS gene signature; [17]) comparing GSEA data of isolated Tfh cells from AITL patients (*n* = 6) versus healthy donor Tfh cells (*n* = 7) and versus public RNAseq data from healthy Tfh cells (Tfh_public, *n* = 7), central memory (Tcm, *n* = 6), effector memory (Tem, *n* = 6), naive (Tn, *n* = 6), regulatory (T reg, *n* = 12) and stem cell memory (Tscm, *n* = 6) T cells. The corresponding GSEA for the OXPHOS signature genes in AITL Tfh is indicated. For all genes with enrichment score >0 (black bars in the pink zone), expression is upregulated in hAITL. Kolmogrov–Smimov (KS) test. **B** Heatmap for GSEA data of genes implicated in mitochondrial biogenesis for the same T-cell fractions as in **A**. The corresponding GSEA for the mitochondrial biogenesis pathways in AITL Tfh indicated in **B** is shown. **C** Mitochondrial pathway analysis for expression data of the same T-cell populations as mentioned in **A** was performed using the KEGG database. Bubble representation (Bubbles size and numbers represent the sample enrichment score (SES), *p* values are indicated in Figure S3C). **D** Mitochondrial biogenesis pathway analysis for gene expression data of the same T cell populations as mentioned in **A** was performed using the Reactome database. Bubble representation (Bubbles size and numbers represent the sample enrichment score (SES), *p* values are indicated in Fig. S3D). **E** FACS analysis of mitochondrial content stained by mitotracker green (MTG) for healthy donor CD4+ T cells and hAITL total CD4+, CD4+ PD-1^low^ and CD4+ PD-1^high^ cells. (mean ± SD, *n* = 3; ***p < 0.001). **F** FACS analysis of ROS content stained by CellROX probe for healthy donor CD4+ T cells, hAITL total CD4+ T cells, CD4+ PD-1^low^ and hAITL CD4+ PD-1^high^ cells. (mean ± SD, *n* = 3; ***p < 0.001).

To confirm the mitochondrial dependence of isolated AITL Tfh cells, we generated affymetrix data for CD4+PD-1 high Tfh cells isolated from 6 different hAITL patient biopsies and for 7 isolated healthy donor Tfh cells. Heatmap GSE analysis revealed a statistically significant upregulation in AITL and healthy donor Tfh subsets for a signature of 52 genes encoding for mitochondrial respiration enzymes (Fig. 3A). This OXPHOS signature GSEA was also enriched for public data available for Tfh cells of 7 healthy donors, while this gene signature was not enriched in other CD4 T-cell subtypes such as central memory (Tcm), effector memory (Tem), naive (Tn) and stem cell memory (Tscm) (Fig. 3A). Interestingly, regulatory T cells (T reg) also was marked by a strong mitochondrial GSEA signature. The dominant OXPHOS signature in AITL and healthy donor Tfh cells was in accordance with a higher expression of genes implicated in mitochondrial biogenesis (Fig. 3B), and the same trend was confirmed for T reg cells. Metabolic KEGG pathway analysis confirmed that AITL and healthy Tfh cells strongly rely on OXPHOS (Fig. 3C). Reactome pathway analysis confirmed in the AITL Tfh subsets a strong upregulation of AITL-specific pathways (Rho GTPase, PD-1, NIK non-canonical NF-kB [8]; Fig. S3A) and dependence on OXPHOS (Fig. S3B). Further analysis revealed in AITL and healthy Tfh increased mitochondrial biogenesis and function (Fig. 3D). Interestingly, also PPARGC1A expression was upregulated, which is a protein essential for de novo generation of mitochondria. Consistent with the above results, we could confirm for hAITL biopsies that their CD4+ PD-1^high^ cells showed significantly higher mitochondrial and ROS content than their CD4+ PD-1^low^ cells (Fig. 3E, F). All together, these data confirm the equivalence between the metabolic requirement of mAITL and hAITL Tfh cells for OXPHOS metabolism.

### AITL-developing mice respond to mitochondrial complex I inhibitors

OXPHOS appeared to be a prominent energy pathway in AITL PD-1^high^ cells, therefore, we next assessed the effects of suppressing this pathway on tumor growth in vivo. Since the plck-GAPDH mice only develop AITL disease at the age of 2 years or older, we established a mAITL transplant model based on NOD/SCIDγ-/- (NSG) mice to allow preclinical drug testing [8, 10].

**Fig. 4:**
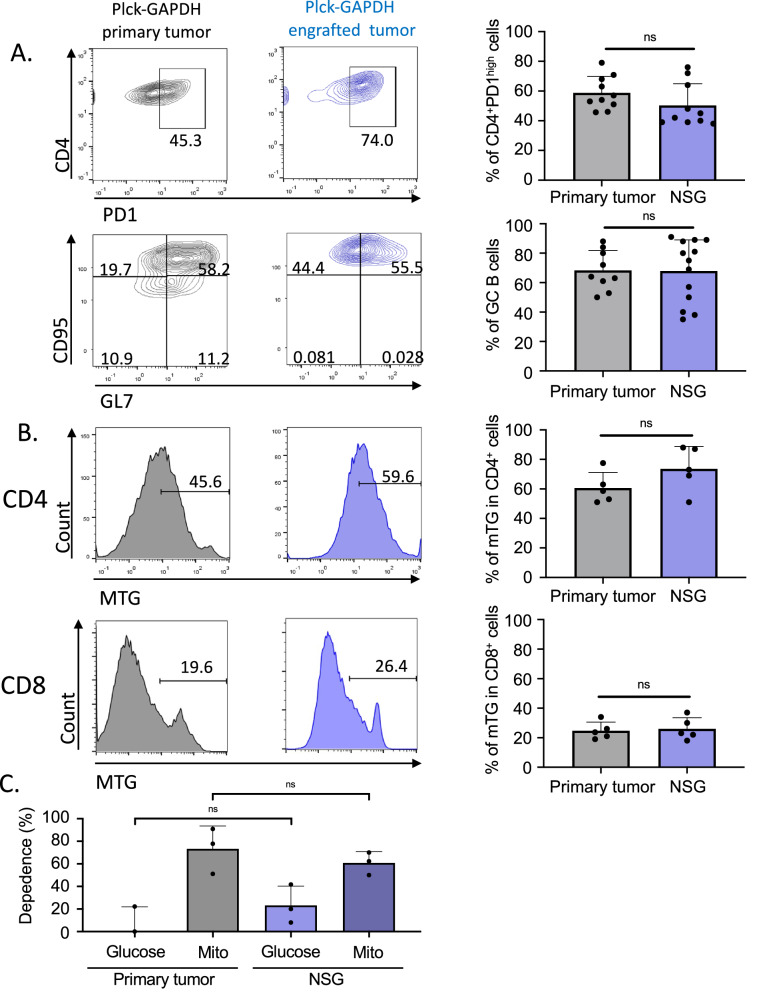
Plck-GAPDH tumor transplanted in NSG mice has the same immune and metabolic phenotype as the primary mAITL lymphoma. Total splenocytes from tumor-bearing Plck-GAPDH mice were transferred to NSG mice, which were sacrificed 8–12 weeks post injection. **A** The % CD4+ DP-1^high^ T cells gated on total CD4+ T cells and GC B cells (CD95 + GL-7+ gated on B220 + CD19+ cells) in spleens from donor plck-GAPDH mice and NSG recipient mice after 8–12 weeks engraftment were analyzed by FACS and summarized in a histogram (mean ± SD; *n* = 3, ns = not significant). **B** FACS analysis of CD8+ and CD4+ T cells of plck-GAPDH donor mice and NSG recipient mice, stained for mitochondrial content by Mitotracker green (MTG) and summarized in histogram (mean ± SD; *n* = 3, ns = not significant). **C** Analysis of the metabolic dependance on glucose or mitochondria for the CD4+ T cells in the spleen of of plck-GAPDH donor mice and NSG recipient mice at sacrifice by SENITH metabolic analysis (mean ± SD; *n* = 3, ns = not significant).

Firstly, we confirmed that mAITL Tfh cells were still addicted to OXPHOS upon engraftment into NSG recipient mice. Next to conservation of CD4+ PD-1^high^ cells and GC B cells (Fig. 4A), SCENITH metabolic analysis confirmed that the strong mitochondrial ATP dependance and high content in mitochondria were maintained in malignant Tfh cells in NSG-engrafted tumors and were equivalent to primary tumors (Fig. 4B, C).

We next treated our mAITL transplant model with metformin, an ETC complex I inhibitor.

**Fig. 5:**
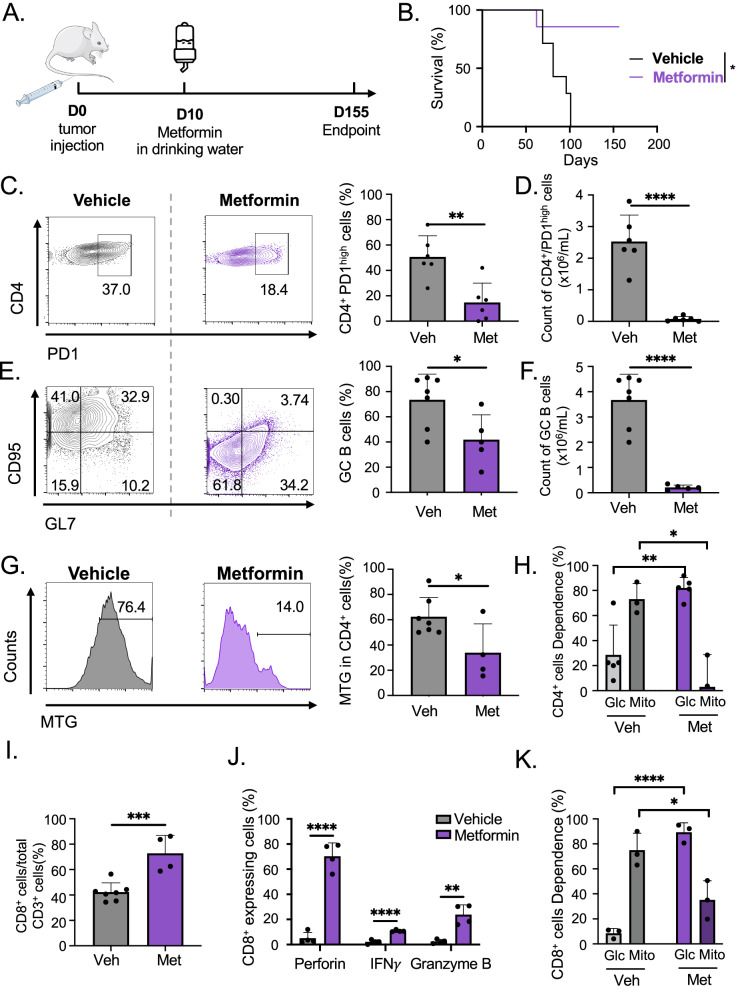
Treatment with Mitochondrial Complex I inhibitor, metformin, prolongs survival of the AITL mouse model. **A** Splenic lymphoma cells from plck-GAPDH mice were injected intravenously into recipient NSG mice (*n* = 13), which were treated with vehicle (*n* = 7) or the complex I inhibitor metformin (*n* = 6) added to the drinking water. Survival curves for mice are shown in **B**. Mice were sacrificed at endpoint or 155 days post-transplant (*p < 0.05, Mantel–Cox test). **C** FACS analysis of percentage of CD4+ PD-1^high^ cells per total CD4+ T cells in the spleen of the indicated treatment groups at sacrifice (mean ± SD, Vehicle (Veh): *n* = 6; Metformin (Met): *n* = 6); **p < 0.01). **D** Total number of CD4+ PD-1^high^ cells in the spleen of vehicle or metformin-treated groups; (mean ± SD, Veh: *n* = 6; Met: *n* = 6); ****p < 0.0001). **E** FACS analysis of percentage of GC B cells (GL-7+ CD95+) on total B cells (gated on CD19 + B220+ cells) in the spleen of the indicated treatment groups at sacrifice (mean ± SD, Veh *n* = 6; Met *n* = 6; *p < 0.05). **F** Total number of GC B cells (GL-7+ CD95+) in the spleen of vehicle or metformin-treated groups, (mean ± SD, Veh: *n* = 6; Met: *n* = 6; ****p < 0.0001). **G** FACS analysis of CD4+ T cells stained for mitochondrial content by Mitotracker green (MTG) in the spleen of the indicated treatment groups at sacrifice (mean ± SD, veh *n* = 7; Met *n* = 4; *p < 0.05). **H** Analysis of the metabolic dependance on glucose or mitochondria for the CD4+ T cells in the spleen of the indicated treatment groups at sacrifice by SCENITH metabolic analysis (mean ± SD, veh *n* = 6, Met *n* = 5; **p < 0.01, *p < 0.05). **I** Percentage of CD8+ T cells per total CD3+ cells in the spleen of the indicated treatment groups at sacrifice (mean ± SD; ***p < 0.001, veh *n* = 7; Met *n* = 5). **J** Splenocytes isolated from metformin or control-treated mAITL engrafted NSG mice were activated for 6 h with PMA/ionomycin in the presence of golgi-stop, then surface stained for CD8 followed by intracellular staining for INFγ, perforin, and granzyme B and analyzed by FACS (mean ± SD, *n* = 4; ***p < 0.001, ****p < 0.0001). **K** Analysis of the metabolic dependance on glucose or mitochondria for the CD8+ T cells in the spleen of the indicated treatment groups at sacrifice by SCENITH metabolic analysis. (mean ± SD, vehicle *n* = 3, Metformin *n* = 3; *p < 0.01, ****p < 0.0001).

We treated the mAITL recipient mice using a clinically relevant protocol by supplementing metformin to the drinking water (Fig. 5A, B). Moreover, we used a dose of metformin equivalent to what is administered orally on a daily basis to diabetic patients. The metformin-treated group showed significant increased survival. A significant decrease in % CD4+ PD-1^high^ per total CD4+ T cells and GC B cells per total B cells in the metformin-treated group was detected and this was even more pronounced when comparing cell counts (Fig. 5C–F). The residual CD4+ T cells in the metformin group lost their dependence on mitochondrial respiration, coinciding with a decreased mitochondrial content (Fig. 5G, H). This metabolic intervention also induced an increase in CD8+ T cells, which gained a strong immune response confirmed by the INFγ, granzyme B and perforin production by these cytotoxic CD8+ T cells in the mAITL preclinical model (Fig. 5I, J). Interestingly, metabolic analysis of the CD8+ T cells in the metformin-treated group showed that they switched to glycolytic metabolism, corresponding to the metabolic requirements of functional effector memory CD8+ T cells (Fig. 5K and [16–19]).

Molina et al. [20] discovered a specific inhibitor of complex I, IACS-010759 (IACS), with a strong effect on acute myeloid leukemia. Clinical trials showed strong toxicity at high IACS doses manifested by neuropathy and acidosis. Therefore, we treated the mAITL mice with low doses of IACS (Fig. S4A). Even at low doses, the IACS-treated group showed a tendency towards improved survival, however, without reaching significance (Fig. S4B). At sacrifice a reduction in malignant CD4+ PD-1^high^ and GC B cells was detected in the IACS-treated mice (Fig. S4C, D), and upon IACS treatment, CD4+ T cells switched from OXPHOS (vehicle) to glucose dependence (IACS) coinciding with lower mitochondrial content compared to vehicle (Fig. S4E, F). Of note, all treatment regimens at the concentration used did not induce toxicities as detected for IACS at low administration in clinical trials.

Summarizing, targeting mitochondrial metabolism in our preclinical mAITL model had a therapeutic effect.

### hAITL Tfh cells respond to the inhibition of mitochondrial ETC complex I

**Fig. 6:**
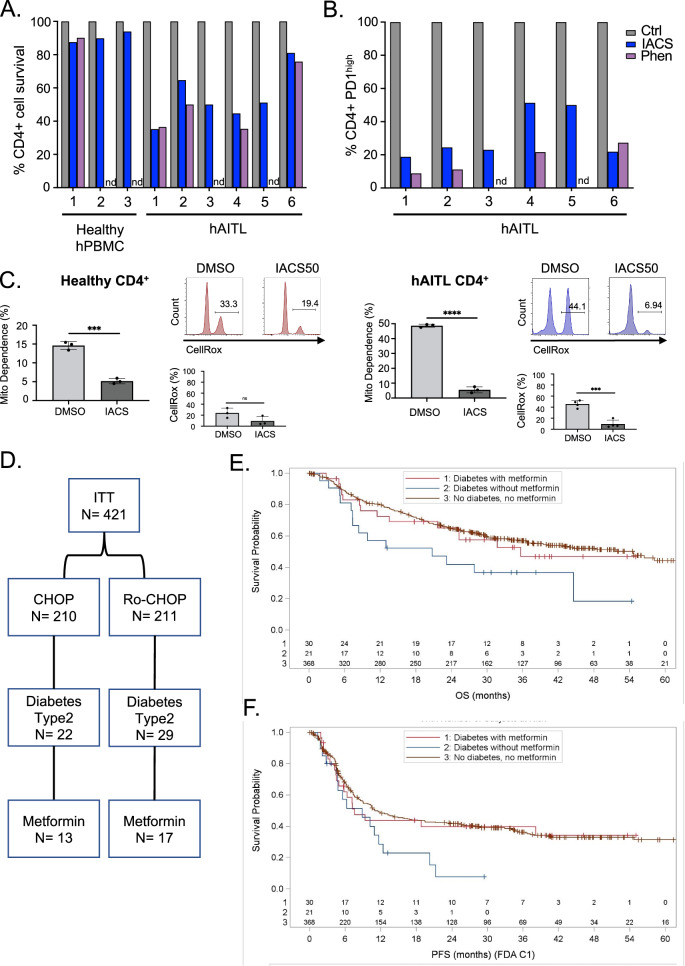
Treatment with mitochondrial complex I inhibitor prolongs survival and progression-free survival of AITL patients compared non-treated ones. Effect of Mitochondrial complex I inhibitors Metformin (Met, **A**) and IACS or Phenformin (Phen) (**B**) on the CD4+ T-cell survival of LN biopsies of 3 and 6 different AITL patients, respectively, compared to healthy CD4+ T cells. Percentages are normalized to corresponding vehicle-treated control cells set at 100%. **C** Analysis of the metabolic dependence on glucose or mitochondrial respiration for the hAITL CD4+ T cells and healthy CD4+ T cells, treated for 48 h with IACS or vehicle by SCENITH metabolic analysis; FACS analysis of healthy donor versus hAITL CD4+ T cells stained for ROS content by CellROX probe treated with IACS or vehicle. The data are summarized in the histogram (mean ± SD, vehicle *n* = 3, IACS *n* = 3; **p < 0.01, ns not significant). **D** Number of patients in the study by Bachy et al. [21] with confirmed type II diabetes treated or not with metformin. **E** Progression-free survival (PFS) for PTCL patients according to diabetes and metformin treatment from the study of Bachy et al. [21]. **F** Overall survival (OS) for PTCL patients according to diabetes and metformin treatment from the study of Bachy et al.

To further emphasize the relevance of ETC complex I inhibition in hAITL, we treated human AITL biopsies containing CD4+ T cells expressing high levels of PD-1 and ICOS (Table S1) with 3 inhibitors: metformin, IACS, or phenformin. Complex I inhibition ex vivo reduced the number of living CD4+ cells in hAITL biopsies while healthy CD4+ T cells remained unaffected (Fig. 6A, B), and this was accompanied by a specific decrease in Tfh CD4+ PD-1^high^ cells for the different biopsies (Fig. S5). We also showed for a representative patient biopsy that inhibition with IACS reduced the mitochondrial dependance of as well healthy as AITL CD4+ T cells. In addition, IACS treatment reduced ROS levels dramatically in the residual hAITL CD4+ T cells (Fig. 6C).

To still reinforce the clinical relevance of inhibiting ETC complex I in PTCL patients, we compared a PTCL patient cohort from the Ro-CHOP study [21], including 421 patients, those who suffered from diabetes type 2 that were treated or not with metformin (Fig. 6D and Table S2 and S3). The diabetic PTCL patients treated with metformin showed an increased PFS as compared to patients treated with alternative drugs (Fig. 6E). In agreement, a similar trend was found for the overall survival (OS) (Fig. 6F). Interestingly, the metformin-treated patient group also showed a reduced level of extranodal AITL lymphoma involvement compared to alternative treatment (Nodules > or equivalent to 2 in Table S2) suggesting an effect on the lymphoma of the metformin treatment.

## Discussion

AITL is a rare lymphoma with very poor survival outcomes and only some adapted treatments have been evaluated in clinical trials such as the use of epigenetic modifiers [22]. Here we show for the first time that human AITL cells preferably rely on mitochondrial respiration. In our murine AITL model, the lymphoma cells used an equivalent metabolic pathway, which allowed us to evaluate ETC inhibitors. Metformin did not only result in a positive outcome on survival of this mAITL mouse model, but also induced the reactivation of cytotoxic CD8 TILs. A more specific complex I inhibitor, IACS, confirmed some therapeutic effects on the mAITL model at doses not toxic in patients [23]. OXPHOS respiration in the malignant CD4+ PD-1^high^ cells was inhibited, resulting in their elimination. Finally, a cohort study revealed a prolonged OS and PFS for diabetic PTCL patients treated with metformin.

Other features of AITL Tfh cells might explain why they are addicted to OXPHOS. Firstly, one factor implicated in Tfh cell differentiation is Bcl-6, which can inhibit glycolysis in Tfh [24, 25]. Moreover, the Bcl-6 locus is hypermethylated in AITL malignant cells, resulting in its overexpression [26], and possibly metabolic reprogramming toward OXPHOS respiration as suggested from our AITL patient data (Fig. S6). Therefore, inhibition of Bcl-6 using « Bcl-6 degraders » might offer an alternative to interfere with AITL cell metabolism. Secondly, engaging PD-1 induces a signaling that limits the glucose uptake and glycolytic flux in T cells [14]. Accordingly, we observed here a reduction in glycolytic ATP production in CD4+ PD-1^high^ cells from mAITL tumors as compared to their PD-1^−^ counterparts, and as shown here PD-1 levels correlated with OXPHOS pathway activation in hAITL. Indeed, we have previously confirmed that anti-PD-1 immunotherapy prolonged the survival of mAITL mice by selectively eliminating the CD4+ PD-1^high^ malignant cells, and restoring CD8 cytotoxicity [8]. Thirdly, the TME has a strong influence on cancer cell metabolism [27]. In AITL, the CD4+ Tfh cells strongly interact with GC B cells, which partially rely on glycolysis for their energy production. The deprivation of glucose by the GC B cells might explain why the Tfh AITL cells are forced to adapt to an OXPHOS metabolism [28]. B cells might suppress T-cell anti-tumor function through metabolic competition. CD8+ T cells in the mAITL TME are not using glucose as their main energy source (Fig. 5K and [29]). Cytotoxic CD8+ T cells rely on aerobic glycolysis for their functions [16, 19, 30]. Indeed, metformin treatment of mAITL mice, relieved CD8 T cells from their ‘exhausted’ state by inducing glycolysis (Fig. 5K). In accordance, Chao et al. [31] and others showed that metformin could rescue T-cell function/motility in vitro and in vivo [32, 33]. Fourthly, the mutational status in AITL might be crucial to identify those patients relying on OXPHOS for lymphoma development. Isocitrate dehydrogenase 1 and 2 (IDH1 and 2), when mutated, can switch cancer cells from glycolysis to OXPHOS [34]. Also, mutations in Ras homolog member A (RhoA), a small GTPase, induced an OXPHOS signature in cancer cells [35, 36]. Importantly, the majority of AITL patients (50–70%) carries a RhoA mutation [37] combined or not with IDH2 mutations. AITL patients with these mutations might particularly benefit from an OXPHOS-targeted intervention and these mutations might be considered as markers for OXPHOS dependance. Our murine AITL model carries an activating RhoA mutation [8], increasing its predictive value for OXPHOS inhibition in patients. Other genetic AITL mouse models harboring TET2 and RHOA mutations in CD4 T cells have been generated [38, 39], or recently, a model combining TET2 and IDH2 mutations [40] might allow to further confirm OXPHOS dependance.

It has become clear that many tumors rely on OXPHOS for their energy, and biomass production [41]. Moreover, cancer stem cells are frequently dependent on OXPHOS [42]. Therefore, subgroups of DLBCL, AML, hepatocarcinoma, and melanomas, might benefit from targeting mitochondrial respiration [43]. Metformin is widely used for the treatment of type 2 diabetes and is associated with lower incidence of cancer [44]. This is attributed to its inhibition of Complex I of the ETC [45] and shows that this drug is safe and effective. In vivo efficacy of metformin was shown in animal models for many different cancers [46]. In some clinical trials, though, outcome in terms of OS and PFS are modest. In our preclinical mAITL model metformin action is not solely relying on the Complex I inhibition in the CD4+ Tfh malignant cells but also on the reactivation of CD8 TIL cytotoxicity. IACS-010759 [20], a more selective complex I inhibitor than metformin, gave impressive results in preclinical studies for cancers reliant on OXPHOS [20]. However, IACS induced severe toxicity at effective doses, such as peripheral neuropathy and acidosis in clinical trials (NCT02882321 and NCT03291938; [23]). Interestingly, in a reverse mouse study, neuropathy was also detected and was attenuated by co-administrations of a HDAC6 inhibitor [47]. At clinically non-toxic doses, IACS showed a tendency towards increased mAITL mouse survival without toxic side effects. We made a compromise by using IACS at a concentration that was not toxic in patients, in our mAITL model. Most probably IACS effectiveness in inhibiting complex I was suboptimal at this dose, but a higher dose might have compromised survival because of its toxic side effects. Other complex I inhibitors (BAY 87-2243, HL156A, ASP4132) performed very well in multiple subcutaneous tumor models, but similar toxicities were noted in clinical trials [48]. This warns for caution in using complex I inhibitors in the clinic and the need for less toxic inhibitors. In contrast, metformin had a significant effect on mAITL mouse survival without toxicity. However, it is well described that metformin has other actions besides inhibiting complex I. It is used as an anti-diabetic drug, which acts directly or indirectly on the liver to lower glucose production, and acts on the gut to increase glucose utilization, reducing glucose levels in the bloodstream [49]. Since glucose can fuel OXPHOS metabolism, its reduced availability might impact AITL neoplastic T-cell survival by this action of metformin. Glucose is most probably not the only source for fueling the mitochondrial metabolism in AITL CD4+ T cells. We detected a significant upregulation of ‘Alanine Aspartate and Glutamate metabolism’, which also contributes to mitochondrial respiration (Fig. 1C). Therefore, interfering with these metabolic pathways might also have an anticancer effect on AITL CD4+ T cells but this will need further exploration. Additionally, we recently demonstrated that neoplastic AITL cells are highly dependent on lipid metabolism. We showed that treatment of our mAITL preclinical mouse model with a fatty acid oxidation inhibitor (FAO), etomoxir, significantly increased their survival [10]. Etomoxir administration exerted an anticancer activity on mAITL cells in vivo and in vitro at levels lower than the one required for ETC inhibition. Indeed, high doses of etomoxir also inhibit complex I of the mitochondrial ETC and exert a stronger effect on neoplastic cell survival, in accordance with our results when using IACs or metformin. Knowing that FAO also contributes to the TCA cycle, and thus, mitochondrial respiration, we might combine FAO inhibition with ETC inhibition to obtain a stronger effect.

Further, we confirmed that the lipid pathway producing phosphatidylcholine was essential for the survival and proliferation of AITL malignant T cells [10]. Targeting the choline lipid pathway using a Chokα inhibitor nearly eradicated all AITL Tfh in our mAITL model and from AITL patient biopsies. This emphasized the therapeutic value of interfering with this specific lipid metabolism. Unfortunately, available Chokα inhibitors are toxic and not approved for use in patients. Hopefully, in the future, new safer drugs interfering with these pathways might lead to new treatments for AITL [10].

A first prospective B-cell lymphoma cohort study showed no evidence that metformin was associated with improved OS in patients with diabetes [50]. In contrast, another meta-analysis of 8 cohort studies, including 8562 patient suffering from non-Hodgkin lymphoma indicated that metformin is associated with improved survival in diabetic patients [46]. However, a better stratification of patients will be required since only some DLBCL rely on OXPHOS [51]. In our cohort of PTCL patients [21] diabetic PTCL patients showed better OS and PFS when treated with metformin. Further follow-up should be conducted in more patients to confirm metformin positive outcome on survival. Overall, our work suggests a new opportunity for the clinical treatment of AITL patients based on the metabolic analysis and intervention in our preclinical mAITL model.

## Methods

### Mice

Mice are bred and maintained under pathogen-free conditions at the local animal facility (C3M, INSERM U1065, Nice, France). Experimental procedures were carried out in compliance with protocols approved by the local ethical and experimentation committee (SBEA, Nice, France, authorization no. 28790-2020121715244498 and B0608820).

#### Plck-GAPDH mice and tumor transplantation into NSG mice

The Plck-GAPDH mouse generation and tumor transplantation are described in ref. [8].

#### Metformin treatment

Two weeks after cell injection, lymphoma-engrafted NSG mice were treated with or without metformin via drinking water (0.2 mg/mL).

#### IACS treatment

Two weeks after lymphoma engraftment, NSG mice were treated with IACS‐010759 [20] by gavage (0,3 mg/kg), as indicated in Fig. S4A. The compound needs to be resuspended in a 0.5% methylcellulose solution (Sigma-Aldrich).

All NSG recipient mice were sacrificed at humane endpoint.

### Cells

#### AITL biopsies and healthy donor PBMCs

Adult healthy blood samples were collected in citrate-dextrose (ACD, Sigma, France) containing tubes. Samples of AITL patients were obtained from the onco-hematology laboratory of the ‘Necker-Enfants Malades’ hospital in Paris (France). The cells were isolated from the lymph node biopsy and frozen in DMSO. All human blood and tissues were obtained after informed consent and approval were obtained by the local ethical commission according to the Helsinki Declaration. Human T cells were isolated from PBMCs using a Pan T-cell enrichment Kit (Miltenyi, #130-096-535) according to manufacturer’s instructions.

### Ex vivo treatment of AITL patient cells

The patient AITL tumor cells or PBMCs were cultured at 5E5 cells/well in RPMI media supplemented with 10% FCS and following human cytokines obtained from Peprotech: 25 ng mL^−1^ IL-6, 50 ng mL^−1^ IL-21, 10 ng mL^−1^ IL-7, 10 ng mL^−1^ IL-15, and 5 ng mL^−1^ IL-2. Treatments: IACS (70 μM), metformin (10 mM), phenformin (300 μM), or vehicle were added to the medium for 48 h. DAPI staining was performed to evaluate cell death by FACS after staining for CD4/CD8/PD-1/CD19. Mitotracker Green (Fisher Scientific; 150 nM) and CellROX Green (Thermofisher) staining were performed after surface staining as described [8]. Antibodies used for phenotyping or intracellular staining by flow cytometry of murine cells are listed in Mondragon et al. [8]

For metabolic analysis and patient cell data analysis, see Supplementary materials.

### Isolation murine immune cells

CD4^+^ T cells from the spleen of WT or plck-GAPDH mice were isolated by negative selection using FITC-coupled antibodies: anti-CD19 (Miltenyi, #130-102-494) anti-B220 (Miltenyi, #130-110-845), anti-CD8b (Miltenyi, #130-111-710), anti-Ter119 (Miltenyi, #130-102-257), anti-NK1.1 (BD Pharmingen, #553164), anti-CD49b (BD Pharmingen, #553857), anti-Ly 6 C (BD Pharmingen, #553127), anti-CD122 (BD Pharmingen,# 554452) and anti-CD11c (BD Pharmingen, #553801), followed by anti-FITC microbead (Miltenyi, #130-048-701) incubation and AutoMACS isolation according to manufacturer’s instructions (Miltenyi). The CD4+ negative T cells fraction was then incubated with anti-PD-1-PE (Miltenyi; #130-111-800) followed by anti-PE microbead (Miltenyi, #130-105-639) incubation and AutoMACS isolation according to manufacturer’s instructions (Miltenyi) to obtain the AITL CD4+ PD-1^high^ and CD4+ PD1- cells for further analysis. WT or Plck-GAPDH CD8+ and CD19+ cells were isolated by and identical procedures using negative microbead selection. The purity of the cells was determined by FACS analysis, and only samples with >90% purity were accepted for further experimentation.

### Flow cytometry and antibodies for murine immune cells

Antibodies used for detailed phenotyping or intracellular staining by flow cytometry of murine T and B cells are listed here and acquired from Miltenyi: CD3 APCcy7 (130-102-306), CD4 FITC (130-102-541); CD8 PEcy7 (130-119-123), B220 FITC (130-110-845), PD-1-PE (130-111-800), CXCR5 APC (130-103-113), ICOS-VB (130-100-639) or BD Pharmingen/ CD19 PE (553786), CD95 VB (562633); INFgamma APC (554413), GL-7 APC (561529) or E-bioscience: Perforin PE (12-9392-82), Granzyme B PEcy7 (25-8898-82).

Staining with MitoTracker® Green (Fisher Scientific; 150 nM) was performed according to manufacturer’s instructions followed by surface Marking before FACS analysis.

For analysis of ROS content by FACS the CellROX Green flow cytometry Assay kit (Thermofisher) was used according to manufacurer’s instructions.

For intracellular staining of Granzyme B, Perforin, IFNγ splenocytes were stimulated for 5 hours in PMA (phorbol 12-myristate-13-acetate; Sigma, # P8139)/ionomycin (Sigma, # I0634) in the presence of Golgi-stop (BD Biosciences, #555029) and upon surface staining (anti-CD4 and anti-CD8) cells were fixed and permeabilized using the Cytofix/Cytoperm kit and protocol (BD Biosciences; #554714).

All stainings were detected using a MACSQuant flow cytometer (Miltenyi Biotec, Paris, France). Analysis of the FACS data was performed using MACSquantify Version 2.11 (Miltenyi) and FlowJo Software.

### Gene expression analysis

#### RNAseq data for plck-GAPDH versus WT spleens

For isolation of RNA and sequencing see Mondragon et al. [8]. RNAseq data were quantified using RSEM software 1.2.25 [52]. with bowtie2-2.2.6 and m38 as the reference genomes. Raw data are submitted on GEO Dataset (GSE121748). For the GSEA were performed using known OXPHOS and glycolytic signatures [13].

#### AITL patient whole lymph node transcriptomics

Public raw data AITL transcriptomes using Affymetrix HGU133 Plus 2.0 microarrays were downloaded from GEO dataset (GSE58445 [11], GSE3526 [12], GSE7307) and ArrayExpress (E-TABM-783 [53]; https://www.ebi.ac.uk/arrayexpress/), normalized together (RMA) and collapsed to HUGO gene symbols using chipset definition files.

#### AITL patient and healthy donor Tfh cell gene expression analysis

Healthy Tfh cells were isolated from healthy donors who got their tonsils removed. T cells were isolated using the Pan T-cell kit (Miltenyi) and purified by FACS sorting for the CD4+ CXCR5 + ICOS + PD-1+ cells. Then cells were processed for RNA extraction. For AITL Tfh cells were isolated from enlarged lymph nodes from patients using the same isolation steps as healthy donor Tfh cells.

RNA was extracted by trizol from purified AITL Tfh cells and Tfh cells from healthy donors. Libraries were prepared and sequenced as described [8]. Affymetrix data are available (GSE232609, confidential Token for access: gnsjgkyozjulnmn) and were quantified using RSEM software 1.2.25 [52] using GRCh38v97 reference genome. These data were compared to public raw data available for healthy donor Tfh, naive, memory, regulatory, and stem cell memory CD4+ T cells downloaded from GEO datasets GSE61697, GSE65010, GSE66384 and GSE71566. Raw files were downloaded and normalized together using RMA methods. GEO number for the Tfh AITL dataset and healthy donor Tfh CD4 dataset: GSE19069, GSE58445 et E-TABM-783 (https://www.ebi.ac.uk/biostudies /arrayexpress /studies/E-TABM-783).

Expression data were normalized with z-score methods when specified and illustrated with heatmaps using R software (3.3.2). Statistical differences were verified using an unpaired two-tailed Wilcoxon signed rank test versus the specified controls. Metabolic pathway analysis was performed using KEGG database: https:// www.ncbi.nlm.nih.gov/pubmed/10592173. For other pathway analysis we used the Reactome database (https://reactome.org/) [54] and MsigDB(https://www.gsea-msigdb.org/gsea/msigdb/) [55].

### Metabolic assays

#### ATP metabolic essay is described in ref. [51]

Briefly, ATP was measured using the Cell Titer Glo Kit (G7570, Promega). Briefly, 20,000 cells were resuspended in 80 μl of the corresponding medium supplemented with 10% FBS and distributed in a 96-well plate. Cells were then treated in triplicates for one hour with PBS (control), or sodium iodoacetate (100 μM) to inhibit glycolysis, or oligomycin (10 μg/ml) to inhibit mitochondrial respiration, or a combination of both drugs to obtain the residual amount of ATP. After one hour of incubation at 37 °C, 100 μL of cell Titer Glo reaction mix was then added to each well for a final volume of 200 μL. Plates were analyzed for luminescence with a Luminoscan (Berthold Technologies). The differences between total ATP and the ATP produced under iodoacetate treatment result in glycolytic ATP contribution. The differences between total ATP and ATP produced after oligomycin treatment result in OXPHOS ATP production.

#### SCENITH metabolic analysis is described in ref. [15]

AITL tumor or WT cells were plated at 1–3 × 10^6^ cells/mL: 100 microliter in 96-well. Murin or human tumor cells were treated during 30–45 min with Control, 2-Deoxy-D-Glucose (DG, final concentration 100 mM), Oligomycin (Oligo, final concentration 1 mM), or a sequential combination of the drugs at the final concentrations before mentioned. As negative control, the translation initiation inhibitor Harringtonine (2 mg/mL) was added 15 min before the addition of puromycin. Puromycin (final concentration 10 mg/mL) is added during the last 15-45 min of the metabolic inhibitor treatment. After puromycin treatment, cells were washed in PBS, and then primary conjugated antibodies against surface markers during 25 min at 4 °C in PBS 1×5% FCS, 2 mM EDTA (FACS wash buffer). After washing, cells were fixed and permeabilized using FOXP3 fixation and permeabilization buffer (Thermofisher eBioscience) following manufacturer instructions. Intracellular staining of puromycin using an Alexa Fluor 647 coupled anti-puromycin monoclonal antibody was performed by incubating cells during 1 h at 4 °C diluted in permeabilization buffer. Cells were then analyzed by FACs (Milteniy, Macsquant10).

### Patient data analysis

Using the data of the 421 patients included in the Ro-CHOP study [21], we aimed to identify (1) the patients with a medical history of diabetes mellitus and (2) the patients treated with metformin. For medical history, all the verbatims were previously coded with the MedDRA terminology and all the concomitant treatments were coded with the WHODrug dictionary.

Patients with diabetes mellitus were detected using the preferred term (PT) containing the character substring “DIAB”. This led to the detection of four types of diabetes: Type 1 diabetes mellitus, Type 2 diabetes mellitus, steroid diabetes, and diabetes mellitus. Patient with history of metformin use were detected using the ATC codes A10BA (Biguanides) and A10BD (Combinations of oral blood glucose lowering drugs). After the run of this algorithm, 3 classifications were obtained: (1) Patients without history of diabetes mellitus (*n* = 368, subgroup “no diabetes, no metformin”); (2) Patients with type 1 diabetes mellitus (*n* = 2, not analyzed because of the small sample size); (3) Patients with type 2 diabetes mellitus (Type 2 diabetes mellitus, steroid diabetes, and diabetes mellitus) (*n* = 51) divided into: (a) Treated by metformin (*n* = 30, subgroup “diabetes with metformin”) and b) Not treated by metformin (*n* = 21, subgroup “diabetes without metformin”). All the reported data (baseline characteristics, diagnosis, medical history, OS, and PFS) were analyzed using descriptive analyses according to the defined subgroups. The quantitative variables were analyzed using usual statistical parameters: sample size, number of missing data, mean and standard deviation (SD), median and interquartile range (IQR), range (minimum from maximum). The categorical variables were analyzed using frequency counts and percentages. Survival analyses were run using Kaplan–Meier method without the Log-Rank test.

### Quantification and statistical analysis

Statistical analysis was conducted using Microsoft excel 2013 and Prism software v6.0 (GraphPad Software, La Jolla, CA, USA). Results are indicated as means ± SD (standard deviation) in the figure legends unless otherwise indicated. For statistical testing of significance a student’s *t* test or ONE way ANOVA was used followed by Tukey range test to assess the significance among pairs of conditions, the test were justified by checking if there was a normal distribution or not; *p* values and number of biological repeats are indicated in the figure legends. A *p* value < 0.05 was considered to indicate statistical significance. For animal experiments, we used the software GPower 3.1 to reduce to the minimum the number of animals per experiment required to obtain statistical relevance. We performed a *t* test with bilateral analysis, which resulted in 8 animals per group. For the animal experiments, we used blinding for group allocation since we could not detect the level of tumor engraftment by blood analysis or another non-invasive method. We excluded analysis of lymphomas for the animals that were found dead; we included only the tissues from animals that were sacrificed because they reached the humane endpoint according to pre-established ethical criteria. Mice survival curves were evaluated using Log-rank test to determine significance. All flow cytometry data shown are representative of at least *n* = 3 reproduced biological repeats and this is indicated in the figure legend. GSEA was performed as described above.

### Supplementary information

Supplemental material

## Data Availability

All RNAseq data and affimetrix data generated or used in this study have been deposited at GEO and are publicly available as of the date of publication. Assession numbers are listed in the methods sections or the supplementary materials. Any additional information to reanalyze the data reported in this paper is available from the lead contact upon request. The data individual participants data used for the retrospective study are shared upon request after validation of a scientific committee.
