# Supplementary material for: Dependence on mitochondrial respiration of malignant T cells reveals a new therapeutic target for angioimmunoblastic T-cell lymphoma
Source: Cell Death Discov. 2024 Jun 19;10:292. doi: 10.1038/s41420-024-02061-9 (PMC11187159; doi:10.1038/s41420-024-02061-9)
Supplement: Supplementary file 1 — Supplemental material [file 41420_2024_2061_MOESM1_ESM.pdf]

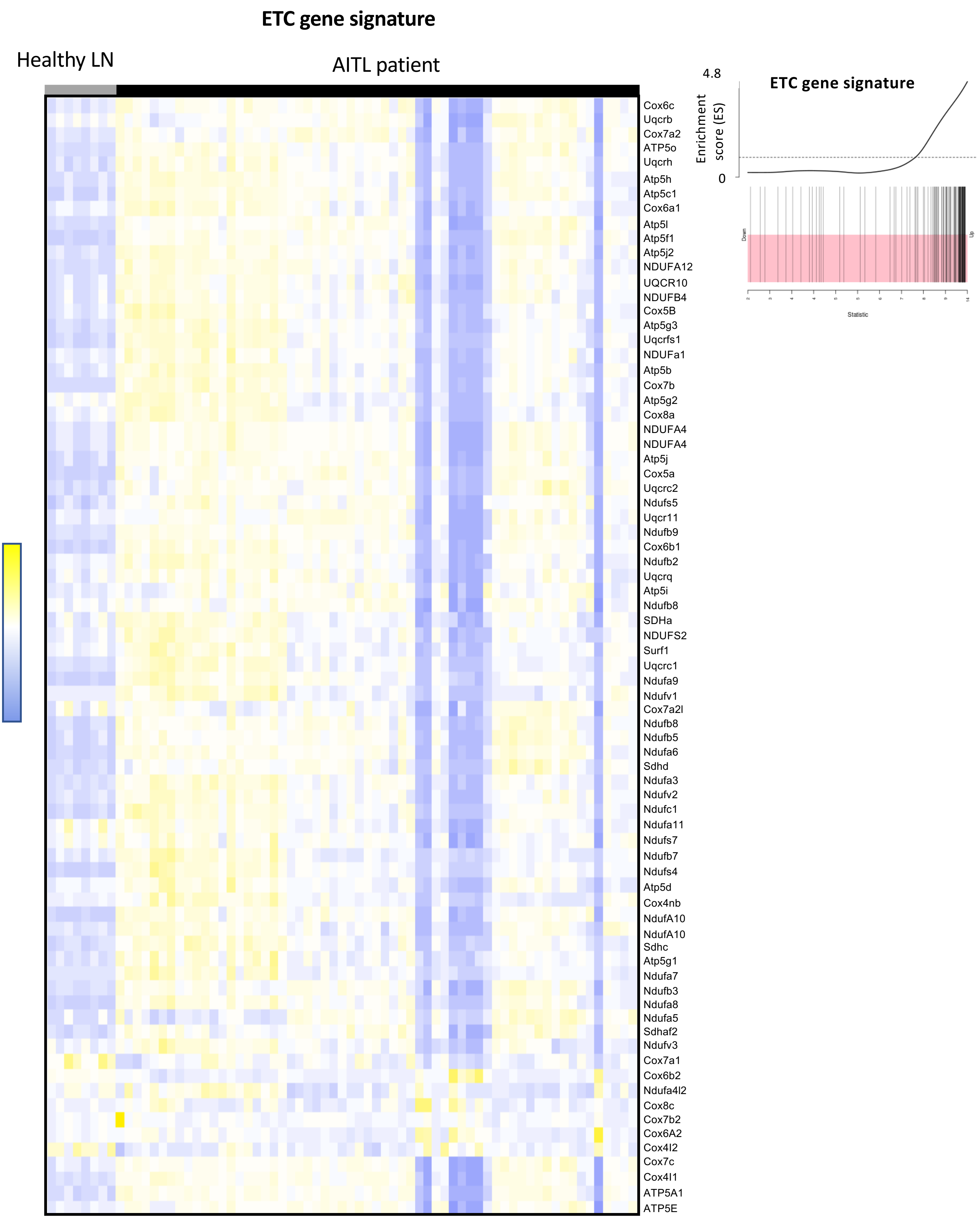

Supplemental Figure 1: **GSEA reveals an upregulated electron transport chain signature in AITL tumors.** Heatmap based on transcriptomic data of 76 genes from a ETC gene signature for AITL patient lymph nodes (LN) (n=60) and healthy LNs (n=8). The corresponding GSEA for the ETC gene is shown. For all genes in GSEA with enrichment score > 0 (black bars in the pink zone), expression is upregulated. Kolmogrov-Smimov (KS) test.

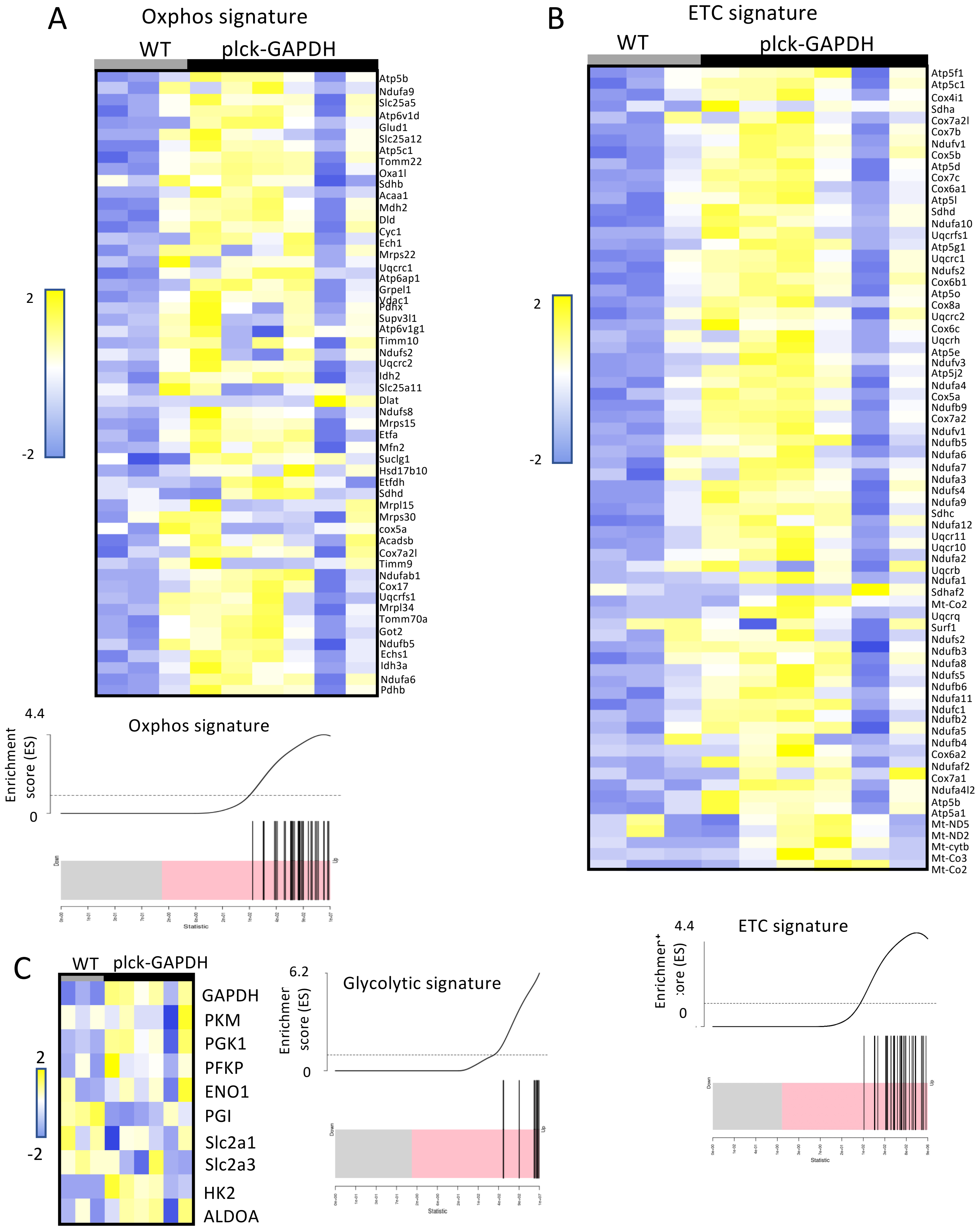

Supplemental Figure 2. Plck-GAPDH AITL tumors show a strong upregulation of oxidative phosphorylation gene signature (OXPHOS).

(A) Heatmap for GEA data for 54 genes implicated in mitochondrial respiration (oxphos gene signature[17]) comparing WT splenocytes and lymphoma cells from plck-GAPDH mice. The corresponding GSEA for the Oxphos gene signature indicated in (A) is shown. (B) Heatmap for GEA data of genes implicated in the mitochondrial electron transport chain (ETC) comparing WT splenocytes and lymphoma cells from plck-GAPDH mice. The corresponding GSEA for the glycolytic gene signature indicated in (B) is shown. (C) Heatmap for GEA data of genes implicated in glycolysis comparing WT splenocytes and lymphoma cells from plck-GAPDH mice. The corresponding GSEA for the glycolytic gene signature indicated in (C) is shown. For all genes in GSEA with enrichment score > 0 (black bars in the pink zone), expression is upregulated. Kolmogrov-Smimov (KS) test.

A

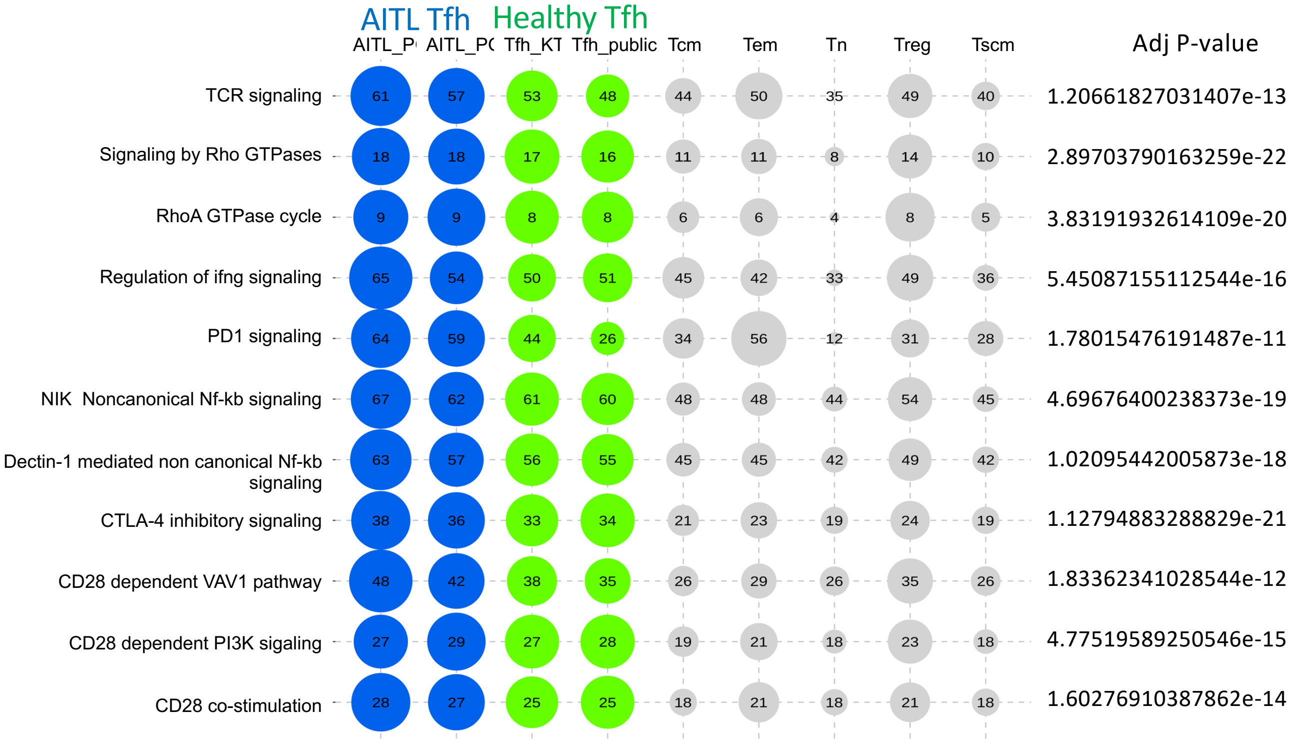

B

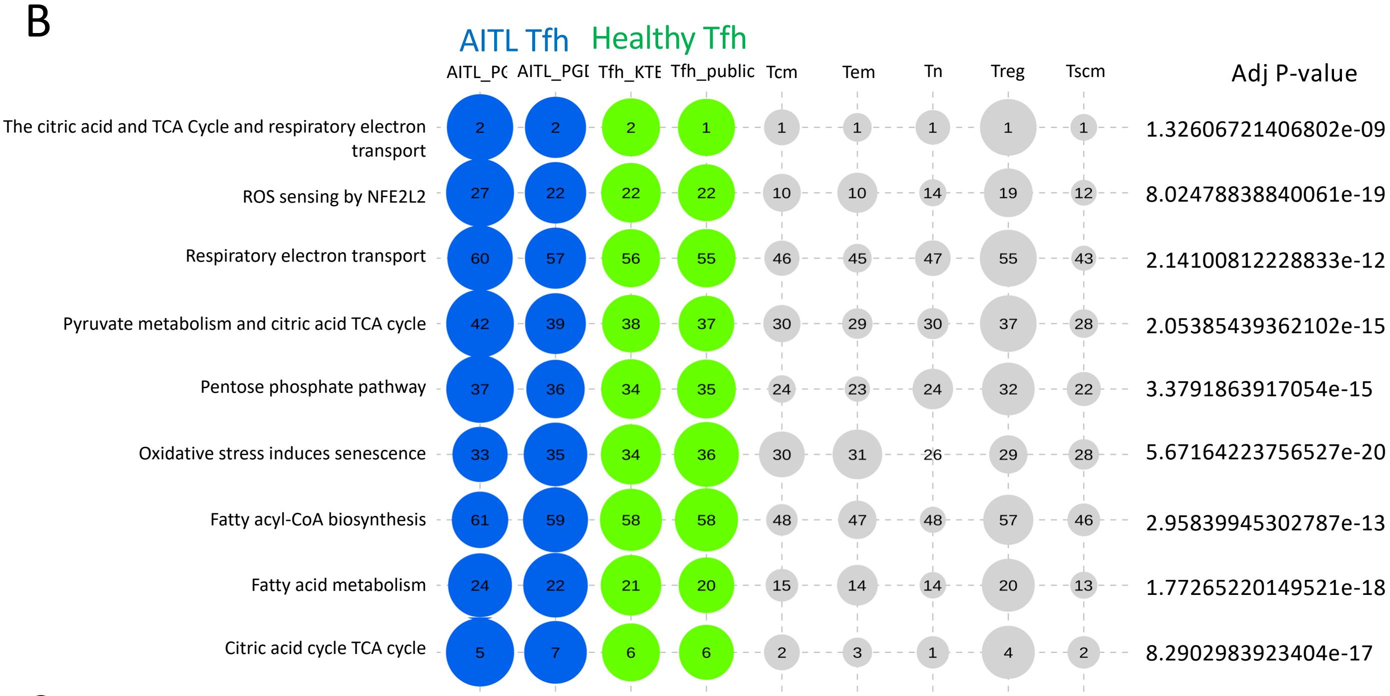

C

Pathway	Adj p-value
Pyruvate metabolism	0.000167075779291942
Pentose phosphate pathway	1.18594765047562e-021
Oxidative phosphorylation	6.04326531465203e-018
mTOR signaling pathway	0.0000000366962745741
Fatty acid metabolism	0.0000000000017437003
Cysteine and methionine metabolism	0.0000000000153234232
Citrate cycle TCA cycle	1.43233907464936e-020
Alanine aspartate and glutamate metabolism	0.0000000000635479619

D

Pathway	Adj p-value
Transcriptional activation of mitochondrial biogenesis	1.76339730231692E-12
Mitochondrial translation	6.04113601447528e-12
Mitochondrial transcription initiation	1.48458401004005e-07
Mitochondrial protein import	1.39819208078732e-07
Mitochondrial biogenesis	1.40686665904405e-17
Cristae formation	3.3791863917054e-15
Activation of PPARGC1a PGC1α by phosphorylation	1.07782903918976e-21

Supplemental figure 3: GEA reveals a strong dependance of AITL malignant T cells on mitochondrial respiration

(A) Specific AITL pathway analysis from RNA-seq data of isolated Tfh cells from AITL lymphoma versus healthy Tfh cells and versus public RNA-seq data from healthy Tfh cells (Tfh_public), central memory (Tcm), effector memory (Tem), naïve (Tn), regulatory (Treg) and stem cell memory (Tscm) was performed using the Reactome database. p-values are indicated.

(B) Mitochondrial pathway analysis from RNA-seq data of the same T cell subfractions in (A) was performed using the Reactome database**.** p-values are indicated

(C) p-Values for the KEGG pathway analysis in Figure 4C and

(D) p-Values for the Reactome pathway analysis in Figure 4D.

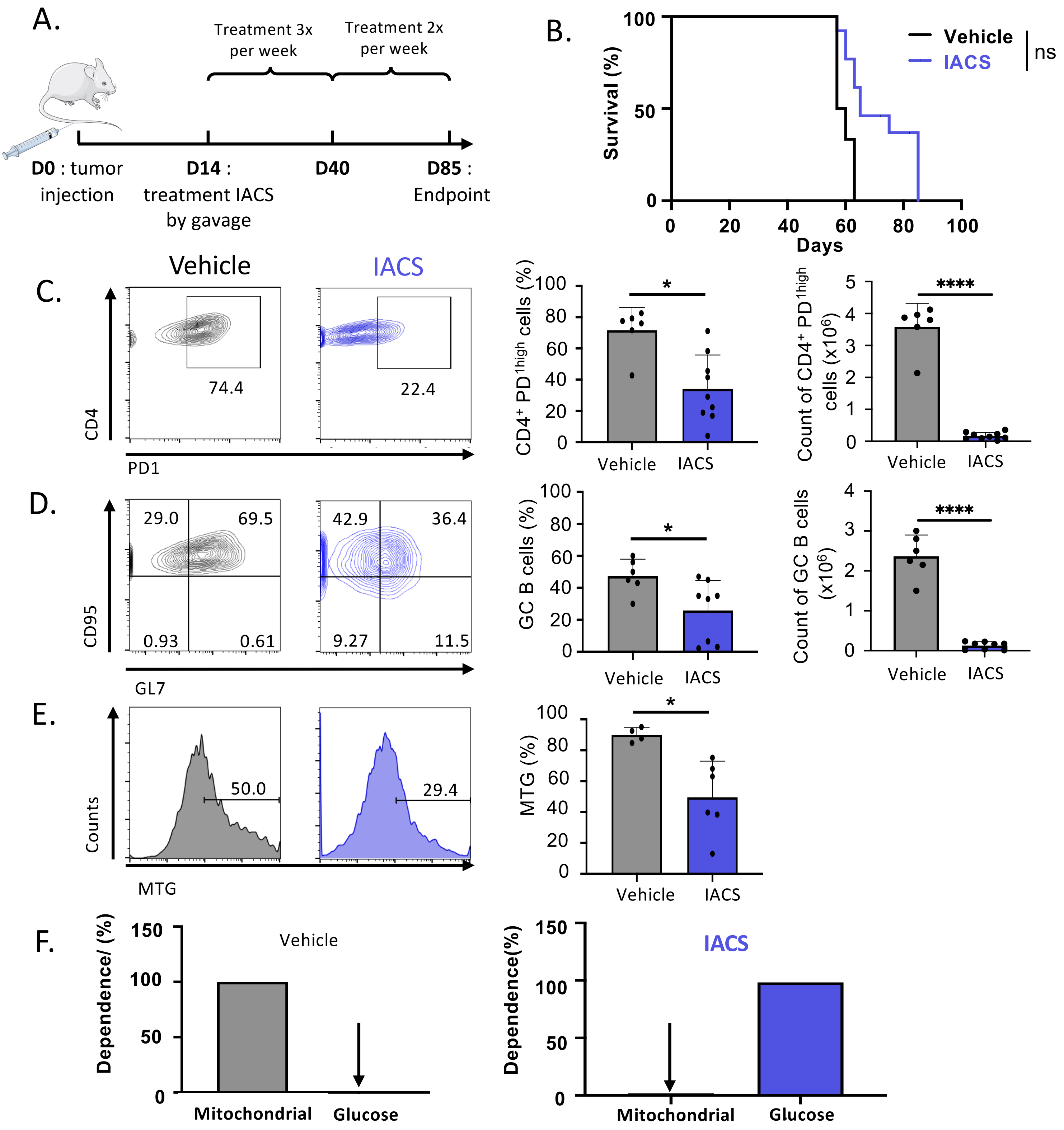

Supplemental Figure 4: Treatment with Complex I inhibitor IACS shows increased survival of the AITL mouse model. (A) Splenic lymphoma cells from plck-GAPDH mice were injected intravenously into recipient NSG mice (n=17), which were treated with vehicle (n=6) or the complex I inhibitor IACS (n=11) by gavage. Survival curves for two treatment groups are shown in (B). Mice were sacrificed at 85 days post transplant (Mantel-Cox test, ns not significant). (C) FACS analysis of % CD4+ PD1^{high} cells on total CD4+ T cells in the spleen for the two treatment groups at sacrifice; data are summarized in the histogram (mean±SD, Veh n=6, IACS n= 9; *p<0.05); the CD4+ PD1^{high} cell counts per spleen are shown (mean±SD, Veh n=6, IACS n= 9; ****p<0.0001). (D) FACS analysis of % GC B cells (GL-7+ CD95+ gated on B220+CD19+ populations) on total B cells in the spleen for the two treatment groups at sacrifice; data are summarized in the histogram (mean±SD, Veh n=6, IACS n= 8; *p<0.05) and GC B cell counts per spleen are indicated (mean±SD, Veh n=6, IACS n= 8; ****p<0.0001). (E) FACS analysis of CD4+ T cells stained for mitochondrial content by Mitotracker green (MTG) in the spleen of the two treatment groups at sacrifice; data are summarized in the histogram (mean±SD, Veh n=4, IACS n=6), *p<0.05) (F) Analysis of the metabolic dependance on glucose or mitochondria for the CD4+ T cells in the spleen of the indicated treatment groups at sacrifice by SCENITH metabolic analysis using FACS.

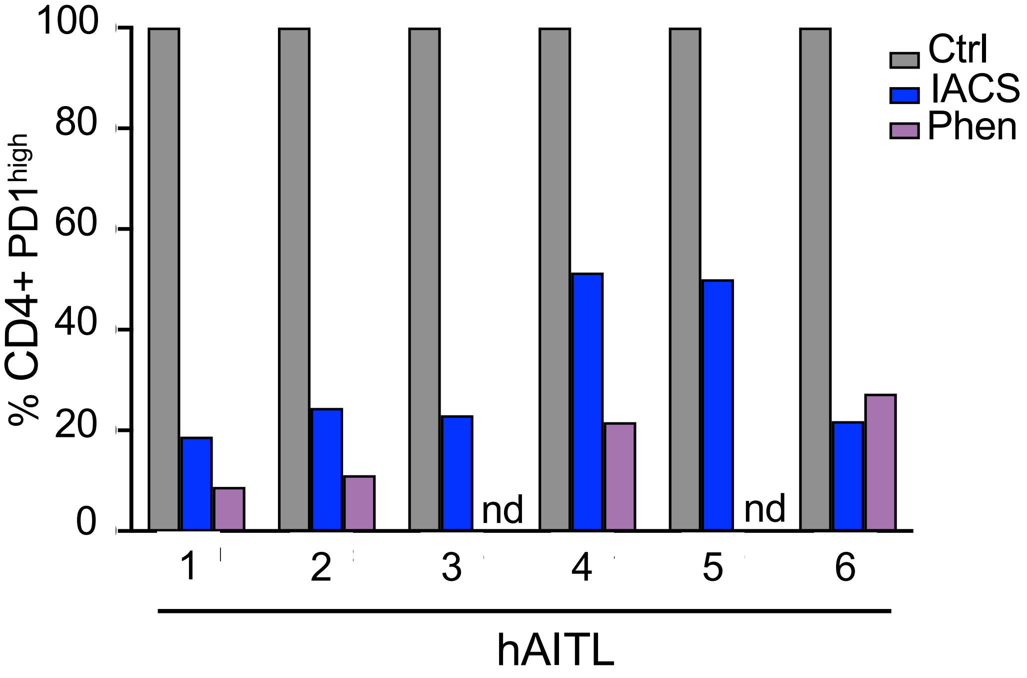

Supplemental Figure 5: Treatment with Mitochondrial Complex I inhibitors eliminates malignant hCD4+ PD1^{high} cells

Effect of Mitochondrial complex I inhibitors (IACS, Phenformin (Phen)) on the % of CD4+ PD1^{high} cells/Total CD4+ cells) in LN biopsies of 6 different AITL patients. Percentages are normalized to corresponding vehicle-treated control cells set at 100%.

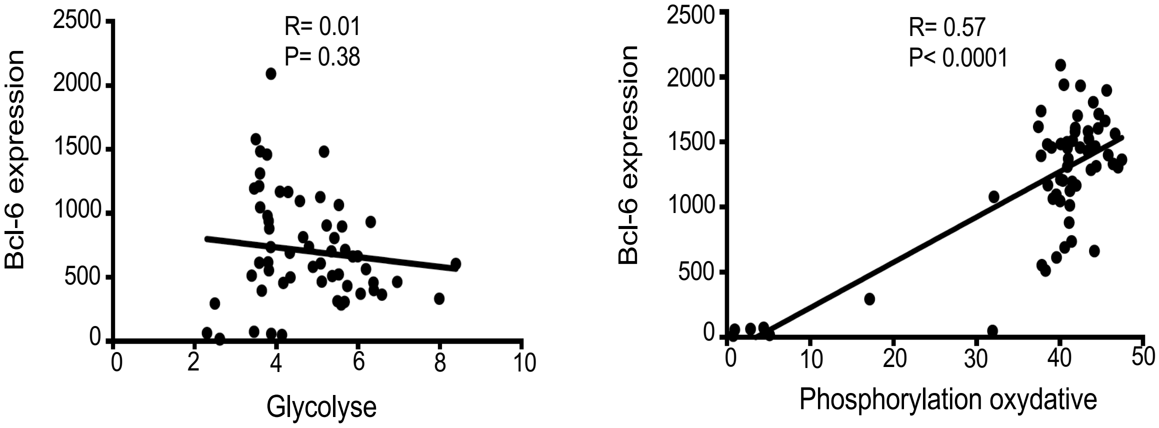

Supplemental figure 6: hAITL tumors show a strong upregulation of OXPHOS signature genes that correlates with an elevated Bcl-6 expression

Correlation of Bcl-6 expression with a glycolytic gene expression signature (A) or with an OXPHOS signature (B) in hAITL tumors.

Supplementary Table 1. Immune phenotype of the T cells in the human AITL biopsies and healthy donor peripheral blood mononuclear cells

PBMC	%CD3+ cells/biopsy	% CD4 cells/total CD3+ cells	% PD1+CD4/ Total CD4+ cells	% ICOS+/ total CD4+ cells
1	80	53	5	3
2	77	50	7	5
3	67	44	11	7
**AITL Patient**	**%CD3+ cells/biopsy**	**% CD4 cells/total CD3+ cells**	**% PD1+CD4/ Total CD4+ cells**	**% ICOS+/ total CD4+ cells**
1	98	93	67	33
2	15	35	71	54
3	60	76	53	71
4	71	70	73	44
5	69	87	60	35
6	98	87	78	57

Supplementary Table 2. Baseline characteristics of the PTCL patients from the clinical study by Bachy *et al*. 2022.

	Diabetes and metformin			
	Diabetes without metformin		Diabetes with metformin	
	N=21		N=30	
**Age**				
N	21		30	
Missing	0		0	
Mean (SD)	69.9 (5.37)		67.1 (6.99)	
Median	71.0		68.0	
Q1 ; Q3	66 ; 73		64 ; 72	
Min ; Max	58 ; 79		50 ; 79	
**Sex**				
Male	15	(71.4%)	21	(70.0%)
Female	6	(28.6%)	9	(30.0%)
**Time since diagnosis (month)**				
N	20		30	
Missing	1		0	
Mean (SD)	1.309 (1.1719)		1.015 (0.5819)	
Median	0.920		0.936	
Q1 ; Q3	0.64 ; 1.72		0.53 ; 1.31	
Min ; Max	0.07 ; 4.63		0.20 ; 2.43	
**Extra-nodal involvement**				
<2	8	(38.1%)	17	(58.6%)
>=2	13	(61.9%)	12	(41.4%)
Missing	0		1	
**Calculated IPI in classes**				
0-2	3	(14.3%)	12	(41.4%)
3-5	18	(85.7%)	17	(58.6%)
Missing	0		1	
**Diabetes mellitus type**				
Type 2	21	(100.0%)	30	(100.0%)
Missing	0		0	

Supplementary Table 3. Diagnosis of the PTCL patients from the clinical study by Bachy *et al*. 2022.

	Diabetes and metformin			
	Diabetes without metformin		Diabetes with metformin	
	N=21		N=30	
**Histological diagnosis in class (reviewed)**				
AITL	7	(36.8%)	14	(46.7%)
OTHER	6	(31.6%)	7	(23.3%)
PTCL-NOS	3	(15.8%)	5	(16.7%)
ALK-NEG ALCL	3	(15.8%)	4	(13.3%)
Missing	2		0	
**Histological diagnosis in classes (local)**				
AITL	6	(28.6%)	12	(40.0%)
PTCL-NOS	11	(52.4%)	10	(33.3%)
OTHER	3	(14.3%)	3	(10.0%)
ALK-NEG ALCL	1	(4.8%)	5	(16.7%)
